# Draft genome sequences of *Brucella melitensis* from human and animal sources in India

**DOI:** 10.1128/mra.00091-24

**Published:** 2024-07-31

**Authors:** Haris Ayoub, M. Suman Kumar, Rishabh Mehta, Prasad Thomas, Himani Dhanze, K. N. Bhilegaonkar, Vibha Singh, Harith M. Salih, Raghavendra G. Amachawadi

**Affiliations:** 1 Department of Veterinary Public Health, ICAR-Indian Veterinary Research Institute, Izatnagar, India; 2 Department of Bacteriology and Mycology, ICAR-Indian Veterinary Research Institute, Izatnagar, India; 3 Department of Clinical Sciences, College of Veterinary Medicine, Kansas State University, Manhattan, Kansas, USA; University of Maryland School of Medicine, Baltimore, Maryland, USA

**Keywords:** *Brucella melitensis*, draft genome, human, animal

## Abstract

We present the draft genome sequences of 23 *Brucella melitensis* isolates derived from human and animal sources across India with genome size predominantly at 3.207 M and uniform GC content (57.24%) across isolates. The accession numbers and detailed sequencing data enhance the utility of this resource for further genomic studies.

## ANNOUNCEMENT

Brucellosis is recognized as one of the world’s leading neglected zoonoses. It is caused by members of the genus *Brucella*, which exhibit a wide host range, with *B. melitensis* being the most virulent member implicated in causing human disease (1–3). The pathogen poses a significant threat to both animal and public health across the globe with an estimated incidence of 2.1 million cases annually (4–6). A total of 23 *B. melitensis* isolates, comprising 20 from human patients and 3 from small ruminants (Sheep) received at the *Brucella* Lab, Division of VPH, ICAR-IVRI, were included in the study. Primary cultivation of all isolates was carried out by inoculating the samples onto Brucella agar and incubating under 10% CO_2_ at 37°C for up to 7 days, as described in reference (7). The identity of the isolates was confirmed using appropriate biochemical tests and species-specific PCR (AMOS PCR) (8), and the reference strain *B. melitensis* 16M was used as a positive control.

Isolates were subcultured in Trypticase Soy agar at 37°C for 42 hours, and genomic DNA was extracted with the QIAamp DNA Mini Kit (QIAGEN) as per the manufacturer’s protocol. Whole-genome sequencing of the extracted DNA was performed using the Illumina MiSeq platform (miBiome Therapeutics LLP, Mumbai, India) generating 150 bp paired-end reads. The quality assessment and preprocessing of the raw reads were done using FastP v0.23.2 (9), and genome assembly was conducted using Unicycler v0.5.0 (10). The assembled genomes were evaluated for completeness using BUSCO v5.4.6 (11), and genome assembly quality was checked with QUAST v5.2.0 (12). NCBI-PGAP v6.6 (13) was employed for genome annotation. The genomes were analyzed for the presence of antimicrobial resistance (AMR) genes using multiple databases, including ResFinder v4.1.5 (14), NCBI AMRFinderPlus v3.11.26 (15), ARG-ANNOT v1.0.1 (16), and CARD v3.1.2 (17). Default parameters were used for all software unless otherwise specified. The draft genome sequences exhibited high completeness and quality, with genomic characteristics, such as genome size, number of contigs, N50, genome fraction, genome coverage, and GC content, being within expected ranges (Table 1). The genomic analysis of the 23 samples revealed overall consistency, with an average genome size of 3.21 Mb, with coverage ranging from 303.4 to 674.6, and a GC content of approximately 57.24%. Notably, the isolate VPH-08-02 showed higher fragmentation with 69 contigs and a unique N50 of 91,417 bp. Isolate VPH-19-03 revealed a lower genome fraction (93.94%) and may represent a unique genome.

**TABLE 1:** Genome assembly metrics and accession number of 23 Indian *B. melitensis* isolates

Lab ID	Host/source	Total reads (M)	Filtered reads (M)	Genome size (Mb)	Genome coverage	No. contigs	N50	Genome fraction	GC content (%)	Genome assembly accession no.	SRA accession no.
VPH-06–01	Sheep/Semen	7.347044	7.15822	3.251	332.5	26	249,280	99.561	57.22	JAYWOX000000000	SRR27558877
VPH-08–01	Sheep/unknown	6.596996	6.410628	3.251	297.8	25	249,280	99.561	57.22	JAYWOY000000000	SRR27558876
VPH-08–02	Sheep/unknown	6.681382	6.527074	3.248	303.4	69	91,417	99.394	57.22	JAYWOZ000000000	SRR27558865
VPH-19–01	Human/bone marrow	11.298194	11.199012	3.207	527.3	25	276,315	99.169	57.25	JAYWPA000000000	SRR27558861
VPH-19–02	Human/unknown	11.943824	11.836264	3.207	557.3	24	293,115	99.168	57.25	JAYWPB000000000	SRR27558860
VPH-19–03	Human/bone marrow	6.416248	6.238506	3.067	307.1	25	249,285	93.937	57.22	JAYWPC000000000	SRR27558859
VPH-19–04	Human/blood	14.560218	14.313068	3.204	674.6	27	293,077	99.105	57.25	JAYWPD000000000	SRR27558858
VPH-19–05	Human/blood	12.62483	12.38699	3.207	583.2	24	293,091	99.171	57.25	JAYWPE000000000	SRR27558857
VPH-19–06	Human/blood	12.559194	12.426234	3.206	585.3	25	293,148	99.143	57.25	JAYWPF000000000	SRR27558856
VPH-19–07	Human/blood	12.068372	11.959162	3.207	563.1	24	293,115	99.168	57.25	JAYWPG000000000	SRR27558855
VPH-20–01	Human/blood	10.706906	10.519896	3.207	495.3	24	293,091	99.171	57.25	JAYWPH000000000	SRR27558875
VPH-20–02	Human/blood	14.377688	14.236112	3.207	670.3	24	293,099	99.168	57.25	JAYWPI000000000	SRR27558874
VPH-20–03	Human/blood	10.443702	10.298964	3.206	485.1	33	193,986	99.135	57.25	JAYWPJ000000000	SRR27558873
VPH-20–04	Human/blood	13.697438	13.511842	3.207	636.2	24	293,100	99.171	57.25	JAYWPK000000000	SRR27558872
VPH-21–01	Human/blood	9.389988	9.257616	3.206	436	25	293,140	99.146	57.25	JAYWPL000000000	SRR27558871
VPH-21–02	Human/blood	12.795494	12.650802	3.205	596	26	249,289	99.143	57.25	JAYWPM00000000	SRR27558870
VPH-22–01	Human/blood	13.931106	13.763442	3.206	648.2	25	293,168	99.146	57.25	JAYWPN000000000	SRR27558869
VPH-22–02	Human/blood	10.566106	10.458546	3.206	492.6	25	293,147	99.146	57.25	JAYWPO000000000	SRR27558868
VPH-22–03	Human/blood	10.114012	9.966226	3.207	469.3	24	293,076	99.166	57.25	JAYWPP000000000	SRR27558867
VPH-22–04	Human/blood	9.959466	9.845878	3.206	463.7	24	293,092	99.165	57.25	JAYWPQ000000000	SRR27558866
VPH-22–05	Human/blood	12.842948	12.69338	3.206	597.8	25	293,164	99.144	57.25	JAYWPR000000000	SRR27558864
VPH-23–01	Human/blood	11.036014	10.940654	3.207	515.1	24	293,091	99.168	57.25	JAYWPS000000000	SRR27558863
VPH-23–02	Human/blood	13.860822	13.745732	3.207	647.2	24	293,091	99.168	57.25	JAYWPT000000000	SRR27558862

The average number of coding sequences was approximately 3,097, accompanied by three rRNA and around 49 tRNA annotations per isolate. Analysis of AMR genes provided insights into the potential resistance profiles of these *B. melitensis* isolates. The AMR gene *B.suis_mprF* was identified in all isolates. The isolates were typed as ST8 on multilocus sequence typing analysis based on MLST 9 (18) and MLST 21 (19) schemas.

This study presents valuable genomic information on *B. melitensis* isolates from India, shedding light on their genetic diversity, and antimicrobial resistance. The data will contribute to a better understanding of *Brucella* genomics.

## Data Availability

This whole-genome sequencing project has been deposited in GenBank under the accession nos. JAYWOX000000000, JAYWOY000000000, JAYWOZ000000000, JAYWPA000000000, JAYWPB000000000, JAYWPC000000000, JAYWPD000000000, JAYWPF000000000, JAYWPG000000000, JAYWPH000000000, JAYWPI000000000, JAYWPJ000000000, JAYWPK000000000, JAYWPL000000000, JAYWPM000000000, JAYWPN000000000, JAYWPK000000000, JAYWPO000000000, JAYWPP000000000, JAYWPQ000000000, JAYWPR000000000, JAYWPS000000000, and JAYWPT000000000. The versions referenced in this paper are JAYWOX000000000.1-JAYWPT000000000.1. The corresponding Illumina reads are available in the Sequence Read Archive (SRA) under the accession number given in Table 1.
